# *In vivo* activity and low toxicity of the second-generation antimicrobial peptide DGL13K

**DOI:** 10.1371/journal.pone.0216669

**Published:** 2019-05-09

**Authors:** Sven-Ulrik Gorr, Craig M. Flory, Robert J. Schumacher

**Affiliations:** 1 Department of Diagnostic and Biological Sciences, University of Minnesota School of Dentistry, Minneapolis, Minnesota, United States of America; 2 Center for Translational Medicine, University of Minnesota Academic Health Center, Minneapolis, Minnesota, United States of America; Christian Albrechts Universitat zu Kiel, GERMANY

## Abstract

Antimicrobial peptides have been evaluated as possible alternatives to traditional antibiotics. The translational potential of the antimicrobial peptide DGL13K was tested with focus on peptide toxicity and in vivo activity in two animal models. DGL13K was effective against *Pseudomonas aeruginosa*, *Staphylococcus aureus* and methicillin-resistant *S*. *aureus* with minimal bactericidal concentrations similar to the minimal inhibitory concentration. The peptide showed low toxicity to human red blood cells and HEK cells with median lethal dose around 1 mg/ml. The median lethal dose in greater wax moth larvae (*Galleria mellonella*) was about 125mg/kg while the peptide caused no skin toxicity in a mouse model. A novel high-throughput luminescence assay was used to test peptide activity in infected *G*. *mellonella*, thus reducing vertebrate animal use. DGL13K killed *P*. *aeruginosa* in both the *G*. *mellonella* model and a mouse burn wound infection model, with bacterial viability 3-10-fold lower than in untreated controls. Future experiments will focus on optimizing peptide delivery, dose and frequency to further improve the antibacterial effect.

## Introduction

Traditional antibiotics are losing their effectiveness due to increasing bacterial resistance. Combined with a decreasing pipeline of novel drugs, it is feared that many critical infections will be difficult or impossible to treat in the near future [1–3]. A recent European estimate attributes 33,100 deaths in 2015 to drug-resistant bacterial infections [4]. On this background, antimicrobial peptides (AMPs) have been evaluated as possible alternatives to traditional antibiotics for over 30 years [5]. Despite initial optimism, several early AMP candidates were in clinical development for over a decade without reaching regulatory approval and it has been suggested that host-derived AMPs lack activity, suffer from nonspecific cytotoxicity, and susceptibility to proteolysis [6, 7].

Recently designed, second generation AMPs have largely addressed these concerns and several are in clinical development [8, 9]. Thus, it is now clear that new design approaches and a more comprehensive understanding of mechanisms of action provide a path towards overcoming the initial limitations. Indeed, second-generation AMPs appear to provide more robust antibacterial activity under in vivo conditions [9, 10].

We have described the design of the peptide GL13NH2, which exhibits LPS binding and bacteria agglutinating activity but is not bactericidal [11–13]. This peptide was modified to increase its positive charge, resulting in the peptide GL13K (now named LGL13K), which exhibits antibacterial and anti-inflammatory activity in vitro and in vivo [13, 14]. An all D-amino acid version of this peptide, DGL13K, exhibits resistance to proteolysis, enhanced activity against Gram-negative and Gram-positive bacteria and the ability to overcome bacterial resistance mechanisms [14, 15]. Table 1 summarizes select properties of these peptides. We recently found that DGL13K is effective against several drug-resistant bacterial strains without causing new bacterial resistance [15]. In this report, we describe the translational potential of DGL13K with focus on peptide toxicity and in vivo activity in two animal models. DGL13K was bactericidal against *Pseudomonas aeruginosa* and *Staphylococcus aureus*, including a methicillin-resistant strain of the latter. Hemolytic activity and human HEK cell toxicity were low, reaching median lethal dose (LD50) at concentrations of 1 mg/ml or above. Similarly, no toxicity was noted on mouse skin while *Galleria mellonella* (greater wax moth larvae) were somewhat more sensitive with an LD50 of around 125 μg/ml. The insect model was used in a high-throughput real time assay to test peptide activity in vivo. DGL13K reduced infection about 4-fold in this model. A similar effect was seen in a mouse skin burn infection model using topical application of DGL13K. Future experiments will focus on optimizing peptide delivery, dose and frequency to improve further these antibacterial effects.

**Table 1:** Properties of GL13 peptides.

Peptide	Sequence	MW	Net Charge	References
GL13NH2	GQIINLKASLDLL-NH2	1396.67 g/mol	+1	[11, 12]
LGL13K	GKIIKLKASLKLL-NH2	1423.87 g/mol	+5	[13, 14]
DGL13K	Gkiiklkaslkll-NH2	1423.87 g/mol	+5	[14, 15]

Sequences shown in single letter code. L-amino acids are shown as upper case letters; D-amino acids are shown in lower case letters. Molecular weight (MW) and Net charge at pH 7 were calculated using the PepCalc calculator from Innovagen (pepcalc.com).

## Materials and methods

### Bacterial strains

A bioluminescent derivate of *P*. *aeruginosa* PAO1, Xen41 was obtained from Xenogen (Alameda, CA; now Perkin-Elmer, Waltham., MA). *S*. *aureus* Xen36, a bioluminescent derivate of America Type Culture Collection strain 49525, was obtained from Perkin-Elmer. Methicillin-resistant *S*. *aureus* USA300 LAC was kindly provided by Drs. Jennifer Nilson and Gary Dunny, University of Minnesota. Bacteria were cultured at 37°C in liquid cultures prepared in Luria broth or Todd Hewitt Broth (THB) (Difco, Franklin Lakes, NJ) and incubated at 200 rpm. Bacteria were plated on Pseudomonas Isolation Agar (Teknova, Hollister, CA) or 1.5% agar prepared in THB and cultured at 37°C.

### Peptides

The peptides LGL13K (GKIIKLKASLKLL-NH2), DGL13K (Gkiiklkaslkll-NH2) and GL13NH2 (GQIINLKASLDLL-NH2) (Table 1) were purchased from Aapptec (Louisville, KY) or Bachem (Torrance, CA). They were synthesized by Fmoc chemistry and the TCA form purified by reverse-phase HPLC (>95% pure). Peptide purity and identity was verified by the supplier by RP-HPLC and mass spectrometry. Lyophilized peptide powders were resuspended at 10 mg/ml in 0.01% sterile acetic acid and stored at 4°C until use. The activity of peptide batches was validated in our laboratory by MIC assays.

### Human cells

Human red blood cells (RBC) (single donor) were obtained from Innovative Research (Novi, MI).

HEK-Blue hTLR4 (HEK), a derivative of the human embryonic kidney cell line HEK293, were purchased (April 2017) from Invivogen (San Diego, CA). The cells were cultured in *HEK medium* consisting of Dulbecco’s Modified Eagle Medium with L-glutamine and 4.5 g/L D-glucose (Gibco, Thermo Fisher), supplemented with 100 Units/ml penicillin, 100μg/ml streptomycin (Gibco), 10% fetal bovine serum (Hyclone) and 1x HEK-Blue selection (Invivogen). Cells were passaged as recommended by the supplier.

### Minimal inhibitory concentration

MIC assays were performed essentially as described for cationic AMPs [16]. A working stock of 10^5^ colony-forming units (CFU)/ml in 100 μl Mueller-Hinton Broth (Difco) or THB was added to 20 μl of a 2-fold serial peptide dilution (concentration range: 167 μg/ml– 0.16 μg/ml) in 10 mM sodium phosphate, pH 7.4 or a 1:10 dilution of phosphate-buffered saline (PBS) in dH2O. Control samples were incubated with phosphate buffer without peptide. The samples were incubated in 96-well polypropylene plates on a lab shaker for 20 h at 37°C. The optical density at 630 nm (OD630) and bioluminescence were determined in a Synergy HT plate reader (BioTek, Winooski, VT). The OD630 was plotted against peptide concentration and the MIC was read as the lowest peptide concentration that prevented any bacterial growth.

### Minimal bactericidal concentration

Five μl aliquots from individual wells of the MIC plates were plated as individual drops on THB or Luria broth agar (*S*. *aureus*) or pseudomonas isolation agar (*P*. *aeruginosa*) plates. The plates were photographed after overnight incubation at 37°C. Images from each plate were uniformly adjusted for brightness and contrast.

### Hemolytic activity assay

hRBCs were diluted 100x in PBS. 180 μl of diluted RBCs were added to 20 μl of a 2-fold serial peptide dilution in 0.01% acetic acid (HOAc) (peptide concentration range 1000–3.9 μg/ml) and incubated in 96-well polypropylene plates on a lab shaker for 1 h at 37°C. The plates were centrifuged 10 min at 10,000 x g and 100 μl of the supernatant was transferred to a polystyrene 96-well plate. The OD at 405 nm (OD405) was determined in a Synergy HT plate reader. Samples that contained 180 μl diluted RBC and 20 μl 0.01% HOAc were used to determine 0% lysis. Hundred percent lysis was determined in samples that contained RBCs that had been diluted 100x in dH_2_O (180 μl) and mixed with 20 μl 0.01% HOAc. Relative lysis of peptide-containing samples (% Lysis) was expressed as [(OD405 with peptide–OD405 without peptide)/OD405 in dH2O] x 100%.

### Cytotoxicity assay

Human cell toxicity was determined by release of lactate dehydrogenase (LDH) upon peptide treatment. HEK-Blue cells (approx. 25,000 cells/well) were plated in tissue culture treated 96-well plates and cultured overnight in complete medium. The cells were then treated with two-fold serial dilutions of peptides diluted in HEK medium (peptide concentration range 1000–0.98 μg/ml) and incubated for 24h at 37°C. Control samples were incubated in HEK medium without added peptide (0% lysis) or in HEK medium with 0.5% Triton X-100 (100% lysis). Fifty μl of the cell culture medium was transferred to a new 96-well plate and mixed with 50 μl LDH reaction mixture according to the manufacturer’s instructions (Pierce LDH Cytotoxicity Assay, Thermo Scientific, Rockford, IL). Plates were incubated at room temperature for 30 min and 50 μl stop solution added to each well. The optical densities at 490 nm and 630 nm were recorded and the net OD [OD490-OD630] calculated as a measure of LDH activity. Percent cytotoxicity was calculated as the [(netOD with peptide–netOD without peptide)/netOD in 0.5% Triton] x 100%.

### *Galleria mellonella* (greater wax moth) larvae

Freshly delivered sixth instar larvae were obtained from local vendors and maintained in sawdust at 4–8°C until use (typically 1–3 days). Larvae generally ranged from 200–300 mg. The larvae were placed in experimental groups of similar average weights.

### Peptide toxicity in *G*. *mellonella*

Larvae were kept cool for immobilization during injections, which were performed with a 50 μl Hamilton syringe in the second rear proleg. Injection volumes were 25 μl/g, adjusted to the nearest integer. Larvae were injected with 0–200 μg peptide per gram bodyweight and maintained at 30°C after peptide injection. Survival was evaluated by movement in response to prodding and the lack of melanization. Control larvae were injected with PBS (buffer control), the peptide GL13NH2, which does not exhibit bactericidal activity [13], or the L-enantiomer of GL13K [13].

### Skin toxicity

Animal procedures were approved by the Institutional Animal Care and Use Committee at the University of Minnesota. Female Balb/c mice were administered buprenorphine SR and anesthetized with isoflurane before being shaved. Twenty-four, 26 and 48 hours post-shave, 300 μl *HPC vehicle* (0.75% hydroxy-propyl cellulose (HPC), 0.1% Triton X-100, 0.1 mM EDTA) with or without 1.0 mg/ml DGL13K peptide was applied to the skin (3 applications/mouse, 4 mice/group). Twenty-four hours after the last application, all mice were euthanized and skin biopsies approximately 2.5 x 2.5 cm were removed and fixed in 10% neutral buffered formalin for histology. Pictures of skin were taken pre- and post-treatment.

### In vivo luminescence

*G*. *mellonella* larvae were injected with *P*. *aeruginosa* Xen41 (1000 CFU/larvae) and placed in individual wells of a 24-well polystyrene plate. Luminescence was recorded in a BioTek plate reader at 30°C for 13 hours when bacteria were in log phase growth. Five μl PBS or DGL13K in PBS (50μg/g) was injected into each larva and luminescence recorded for 7 hours at 30°C. Surviving larvae were euthanized at -20°C.

### Burn wound infection model

Female Balb/c mice were given analgesia and anesthetized as described above. The mice were then burned for 2 seconds with a 1.5 cm diameter brass rod that was heated in a 95°C water bath. A 2 sec exposure generated a second degree burn, which was verified by histology of skin biopsies. Twenty-four hours after the burn, *P*. *aeruginosa* (10^5^ CFU in 50 μl PBS) were topically applied to the burns. At 2 and 6 hours after the inoculation, 300 μl DGL13K at 1.0 mg/ml in HPC vehicle, HPC vehicle without peptide, or triple antibiotic was applied to the burn wounds (5 mice/group). Bioluminescent imaging to quantify metabolically active *P*. *aeruginosa* at the wound site was carried out in an IVIS Spectrum In Vivo Imaging System (University of Minnesota Imaging Center) at 6 hours (just prior to antibiotic treatment) and 24 hours after inoculation. Mice were humanely euthanized after imaging.

### Statistical analysis

Sample sizes and number of experimental replicates are provided in the figure legends. Statistical analysis was performed as described in the figure legends, using Graphpad Prism v. 6.07 (Graphpad Software, La Jolla, CA).

## Results

### Antibacterial activity

We have previously reported the antibacterial activity of LGL13K and DGL13K against Gram negative and Gram-positive bacteria [13–15]. The activity of LGL13K and DGL13K was confirmed against Gram-negative *P*. *aeruginosa*, whereas only DGL13K was active against Gram-positive *S*. *aureus* and a methicillin-resistant strain of *S*. *aureus* (**Table 2**). For comparison, we chose an AMP (polymyxin B) that is in clinical use, but which is known to lack activity against Gram positive bacteria (Table 2). Thus, the new AMP DGL13K exhibits a broader spectrum than either LGL13K or the clinically relevant AMP polymyxin B.

**Table 2:** Minimal inhibitory concentrations.

Species > Strain >	*P*. *aeruginosa* Xen41	*S*. *aureus* Xen36	*S*. *aureus* USA300
Peptide	MIC (N)	MIC (N)	MIC (N)
LGL13K	10.4 μg/ml (13)	83.3 μg/ml (10)	> (5)
DGL13K	5.2 μg/ml (17)	2.6 μg/ml (11)	5.2 μg/ml (5)
Polymyxin B	0.65 μg/ml (7)	41.7 (4)	83.3 μg/ml (2)

*P*. *aeruginosa* Xen 41, *S*. *aureus* Xen 36 and *S*. *aureus* USA300 (MRSA) were tested against LGL13K, DGL13K and polymyxin B. Median MIC values were calculated from MIC curves based on 4–7 (*P*. *aeruginosa* Xen41); 3–5 (*S*. *aureus* Xen36) or 1–2 (*S*. *aureus* USA300) independent experiments (N as listed). **>** indicates that MIC was above the tested peptide concentration range.

**Fig 1:**
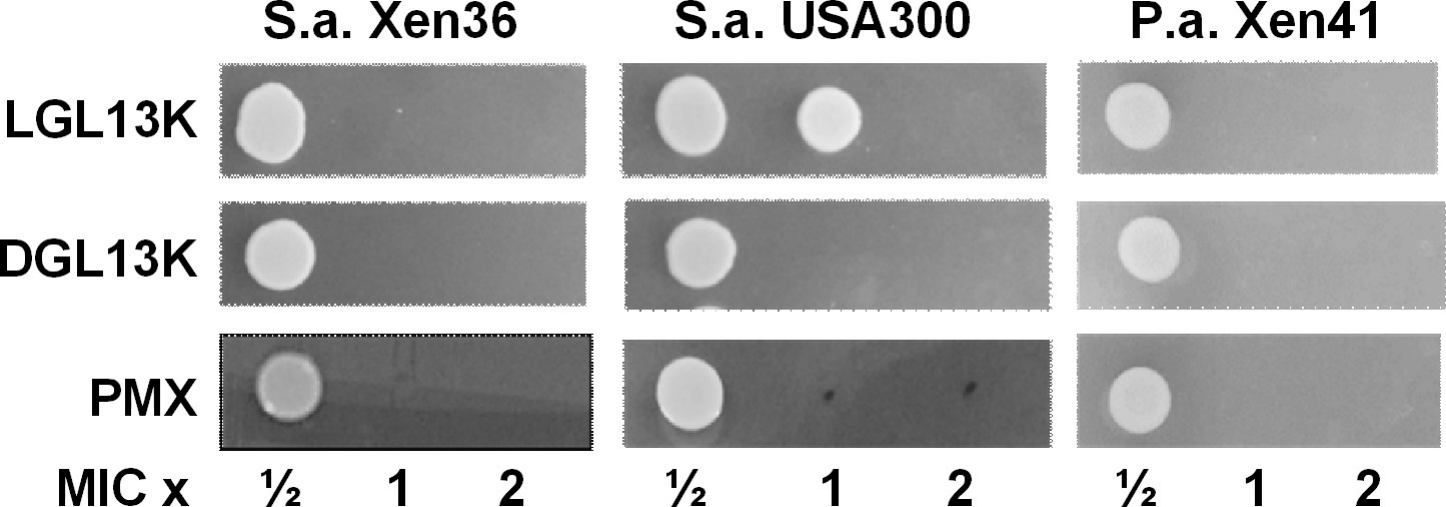
Minimal bactericidal concentrations. *S*. *aureus* Xen36, USA 300 and *P*. *aeruginosa* Xen41 were tested against LGL13K, DGL13K and polymyxin B (PMX). For each peptide, aliquots (5 μl) of overnight cultures at ½, 1 or 2 x MIC were plated as individual drops on agar and cultured overnight. The figure shows the colonies cultured from each drop and is representative of 4–9 (Xen36), 2–4 (USA 300) and 4–7 (Xen41) MIC series.

MBC assays were used to determine if the lack of bacterial growth in the MIC assay was due to bacteriostatic or bactericidal activity. The former would allow regrowth of bacteria upon plating of samples on agar without added peptide. Bactericidal activity would lead to a lack of regrowth on agar lacking the peptide. To determine if the GL13K enantiomers were bactericidal, aliquots of the MIC assays were plated (as 5 μl drops) and cultured on agar. Surviving bacteria were visualized as a bacterial colony growing from the aliquoted drop. Fig 1 shows that the peptides were bactericidal against *S*. *aureus* at 1-2-fold MIC and killed *P*. *aeruginosa* at the MIC.

### In vitro toxicity

**Fig 2:**
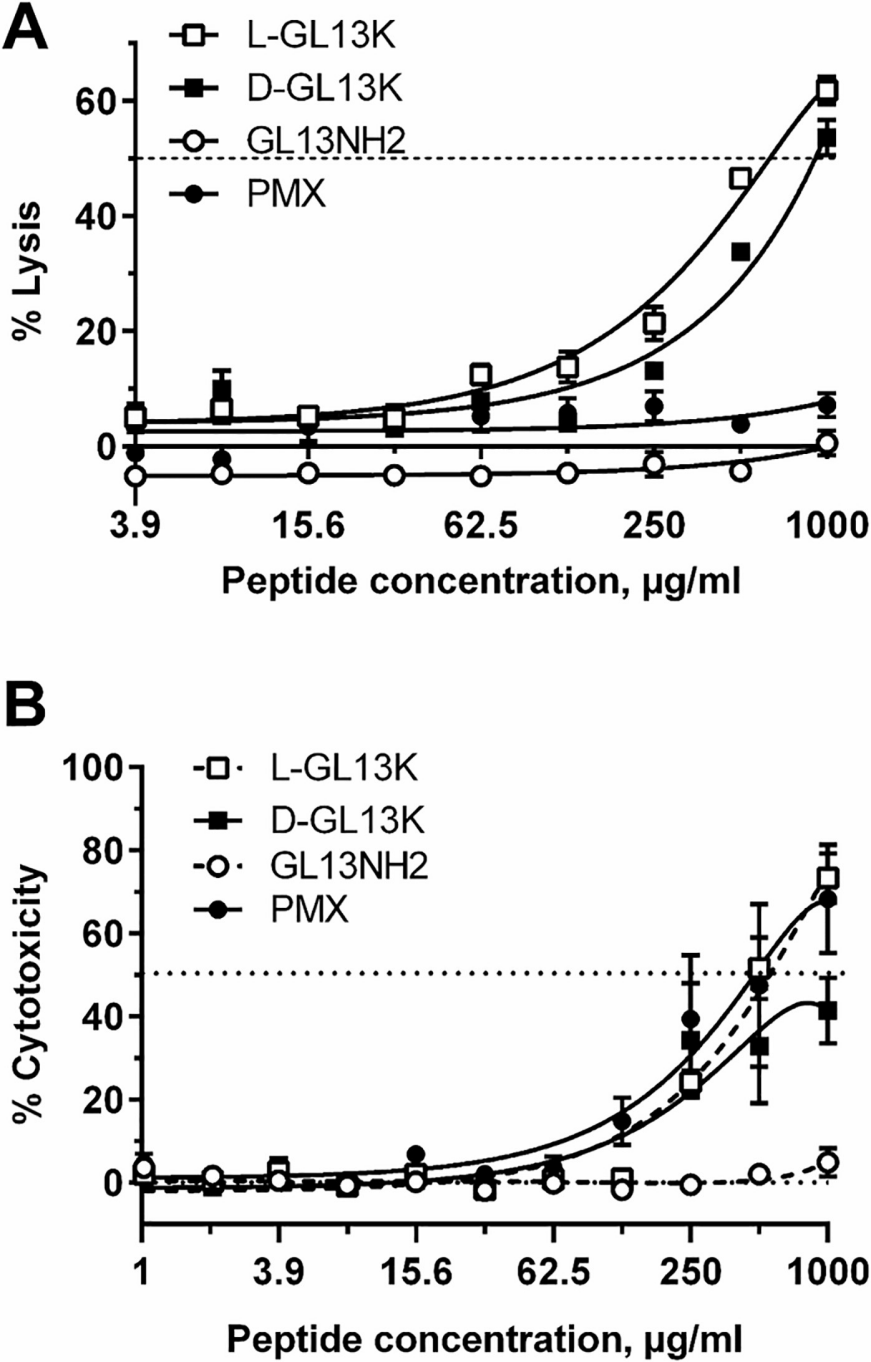
Peptide-induced lysis of human cells. **[A].** Human red blood cells (1%) were incubated with the listed peptides at the concentrations (μg/ml) indicated (PMX–polymyxin B). Released hemoglobin was quantitated spectrophotometrically and expressed as % lysis relative to dH2O (100% lysis). 0% lysis represents hemoglobin released by cells incubated in PBS without peptide. Data from two independent experiments were fitted to polynomial dose-response curves and are shown as mean ± SEM (N = 7). The dotted line indicates 50% lysis (LD50). **[B]:** HEK cells were treated for 24h with each peptide at the concentrations indicated (μg/ml). LDH release (% cytotoxicity) in peptide-treated samples was expressed relative to samples treated with 0.5% Triton X-100 (100% lysis). 0% lysis represents samples incubated in medium without added peptide. Data from two independent experiments were fitted to polynomial dose-response curves and are shown as mean ±SEM, N = 4. The dotted line indicates 50% lysis (LD50).

To determine if the GL13K enantiomers show the necessary selectivity for bacterial cells, their toxicity against human red blood cells (hemolysis) was determined [17]. Dose response curves revealed LD50 values around 0.5 and 1 mg/ml for LGL13K and DGL13K, respectively (Fig 2A). The non-bactericidal GL13NH2 peptide, which is less cationic than GL13K (Table 1), appears to slightly prevent cell lysis, as reflected by the negative lysis values compared to PBS alone.

Toxicity of the GL13K enantiomers against human cells was similarly determined by recording release of LDH as a measure of cell damage after 24 h exposure to serial dilutions of each peptide. LGL13K reached 50% release of LDH at about 0.5 mg/ml, while the LD50 for DGL13K was above 1 mg/ml (Fig 2B). The antimicrobial control peptide polymyxin B showed low hemolytic activity but its cytotoxicity was similar to that of LGL13K against HEK cells (Fig 2A and 2B).

### In vivo toxicity

**Fig 3:**
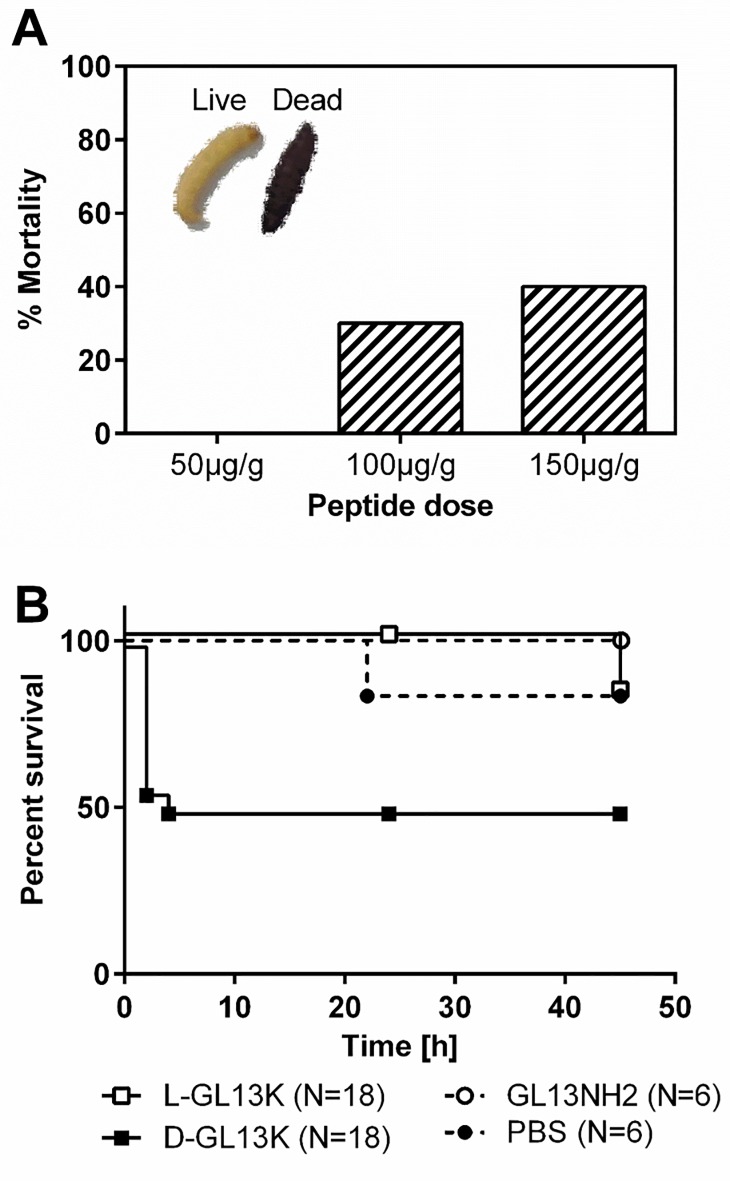
Peptide toxicity in *G*. *mellonella*. [**A].** Dose response of DGL13K after 18 h incubation with DGL13K at the concentrations shown (N = 10 per group). Mortality was assessed as absence of movement upon prodding and heavy melanization (inset, right). [**B].** Survival curves for *G*. *mellonella* treated with 125 μg/g of LGL13K (open squares), DGL13K (closed squares), GL13NH2 (open circles) or buffer control (PBS, closed circles). All larvae were injected with 25 μl per gram body weight. Data from two experiments, N is listed for each peptide.

Larvae of *G*. *mellonella* (greater wax moth) have been used extensively as a convenient animal model for toxicity and infection studies, e.g. [18–22]. Due to the large number of larvae that can be monitored simultaneously, they lend themselves to high-throughput approaches and real-time assays [22]. Larvae were injected with 50–150 μg/g of DGL13K. Viability was assessed at 18h by movement upon prodding and absence of melanization (Fig 3A). Based on these results, the time course of mortality was determined for LGL13K and DGL13K at 125 μg/g larva weight. Fig 3B shows that DGL13K resulted in 50% mortality, consistent with the results of Fig 3A, while LGL13K showed no significant mortality (Fig 3B). Similarly, a less cationic control peptide, GL13NH2 (Table 1), which is not bactericidal [12, 13], was not toxic in this assay (Fig 3B).

**Fig 4:**
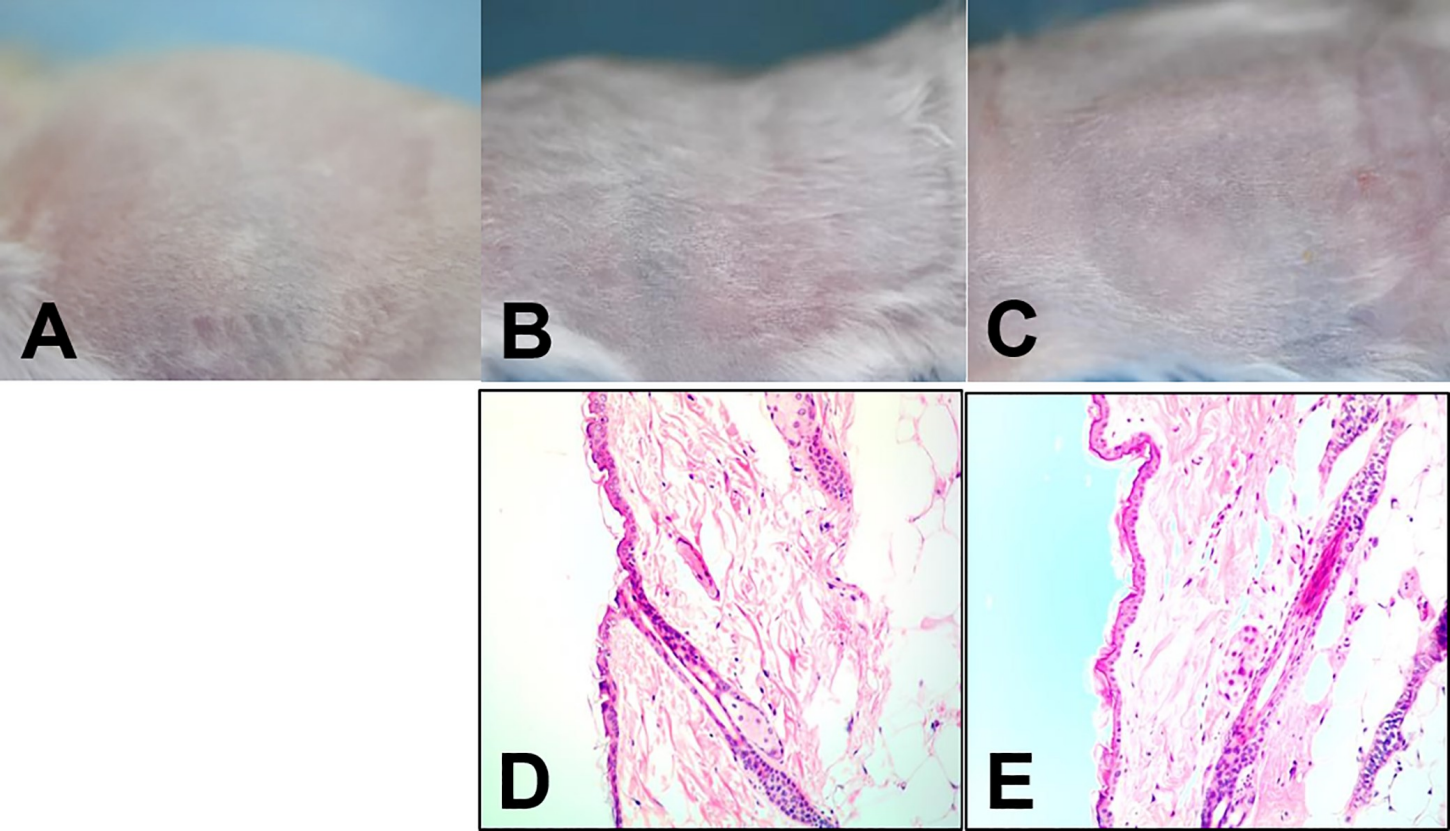
Skin toxicity. Top row: Skin surface before topical treatment [**A]**, and 24h after the last treatment with vehicle [**B]** or vehicle and 1 mg/ml DGL13K [**C]**. Bottom row: Histology of skin biopsies 24h after the last treatment with vehicle [**D]** or vehicle and 1 mg/ml DGL13K [**E]**. Each image is representative of 4 mice.

In preparation for topical application of DGL13K, the peptide was formulated in 0.75% HPC containing 0.1% Triton X-100 to enhance solubility and 1 mM EDTA, which enhances peptide activity in physiological buffers (Jordan and Gorr, unpublished) [23]. Since either the vehicle components or the peptide could cause skin irritation (redness edema), we tested these on intact skin (Fig 4). When compared to untreated mouse skin (Fig 4A), the peptide formulation caused no noticeable irritation (redness, edema) 24 hours after three applications of vehicle alone (Fig 4B) or vehicle with peptide (Fig 4C) over a 48 hour period. Similarly, histopathology of skin biopsies did not reveal inflammatory infiltration or other pathology in the corresponding skin biopsies (Fig 4D and 4E).

### In vivo activity of DGL13K

**Fig 5:**
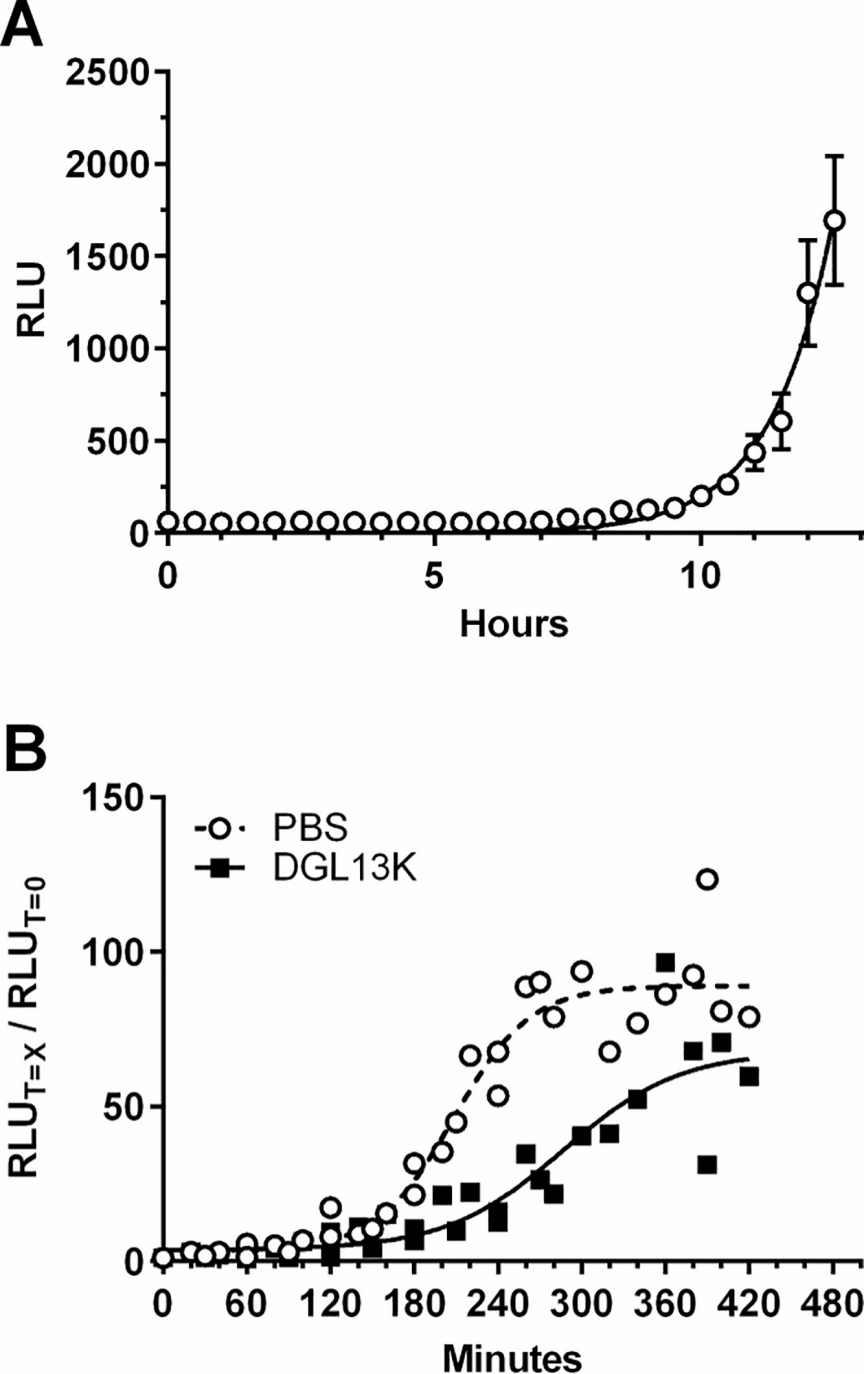
Infection of *G*. *mellonella*. **[A].** Bacterial infection of *G*. *mellonella* larvae monitored by luminescence (relative light units—RLU) in live larvae. Data were fitted to an exponential growth curve and are expressed as mean ± SEM, N = 24. The experiment was repeated with similar results. [**B]**. Effect of DGL13K (50 μg/g; closed squares) and buffer control (PBS) (open circles) on bacterial growth in live larvae. The RLU at each time point (T = X min) was expressed relative to the RLU at T = 0 min for that experimental group. Data from two independent experiments each with 12 larvae per group were fitted to sigmoidal curves (Goodness of fit: PBS R^2^ = 0.94; DGL13K R^2^ = 0.83). Each point represents the mean of 12 larvae.

The *G*. *mellonella* in vivo model was used to test the activity of DGL13K in a high-throughput, real-time assay [22, 24]. Larvae were each injected with 1000 CFU of *P*. *aeruginosa* Xen 41 and luminescence monitored in live larvae (Fig 5A). When bacterial growth reached log-phase, the larvae were injected with DGL13K and bacterial luminescence monitored. Fig 5B shows that DGL13K significantly inhibited continued growth of *P*. *aeruginosa* in live larvae. The plateau of luminescence after 4–5 h incubation coincided with the eventual death of the larvae.

**Fig 6:**
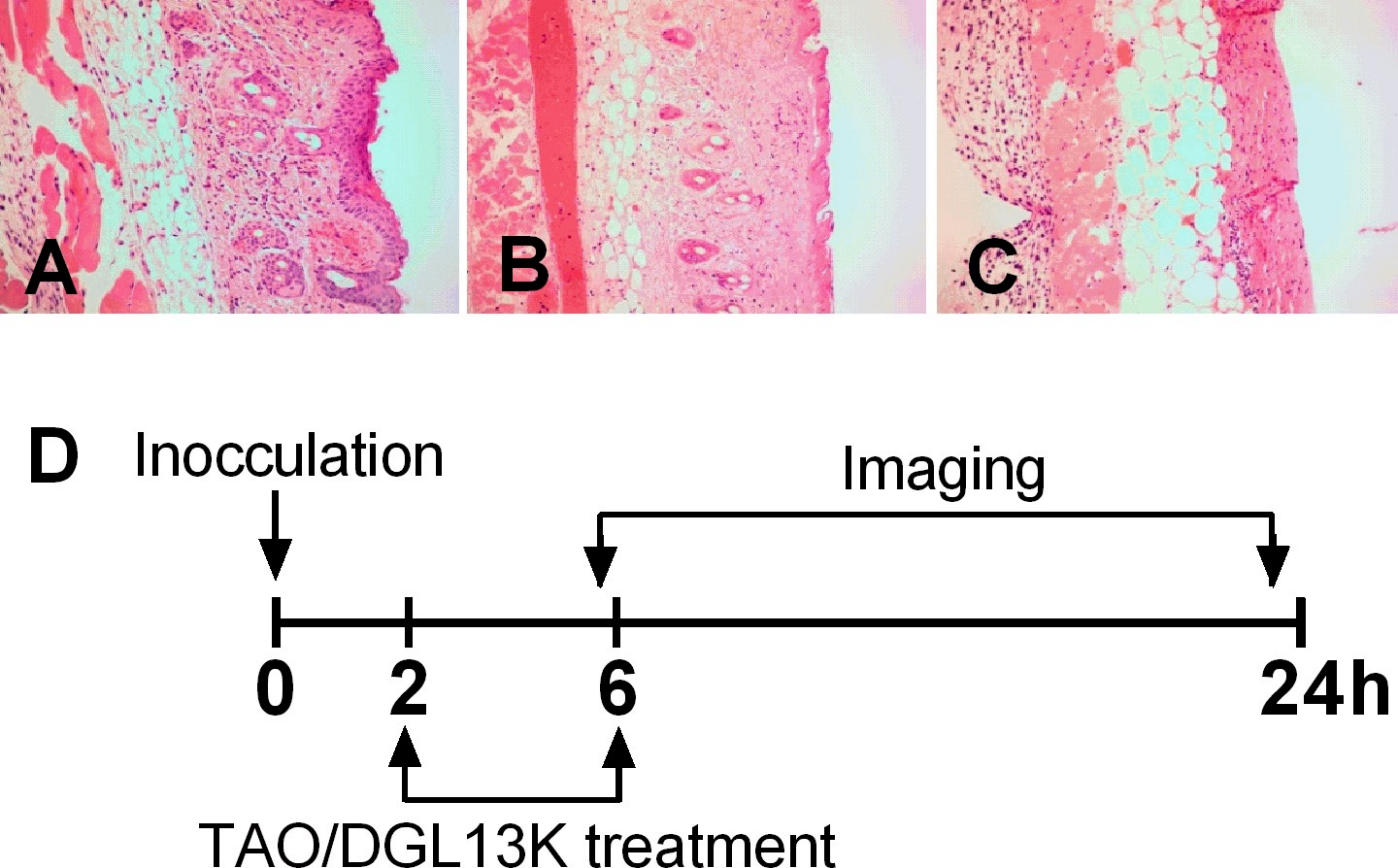
Mouse burn and treatment scheme. [A] untreated mouse skin (H&E stain); [B] skin burned for 2 sec with a brass rod heated to 95°C; [C] skin burned for 4 sec with a brass rod heated to 95°C. [D] Second degree burns were created as in panel B and 24 hours later inoculated with 10^5^ CFU of *P*. *aeruginosa* Xen41 (0 hours). The wounds were treated topically 2 hrs and 6 hrs after inoculation with HPC vehicle, triple antibiotic ointment (TAO) in petrolatum or DGL13K (1mg/ml) in HPC vehicle. The mice were imaged at 6hours, just before the second antibiotic treatment and 24 hours after inoculation (18 hours after the second antibiotic treatment).

**Fig 7:**
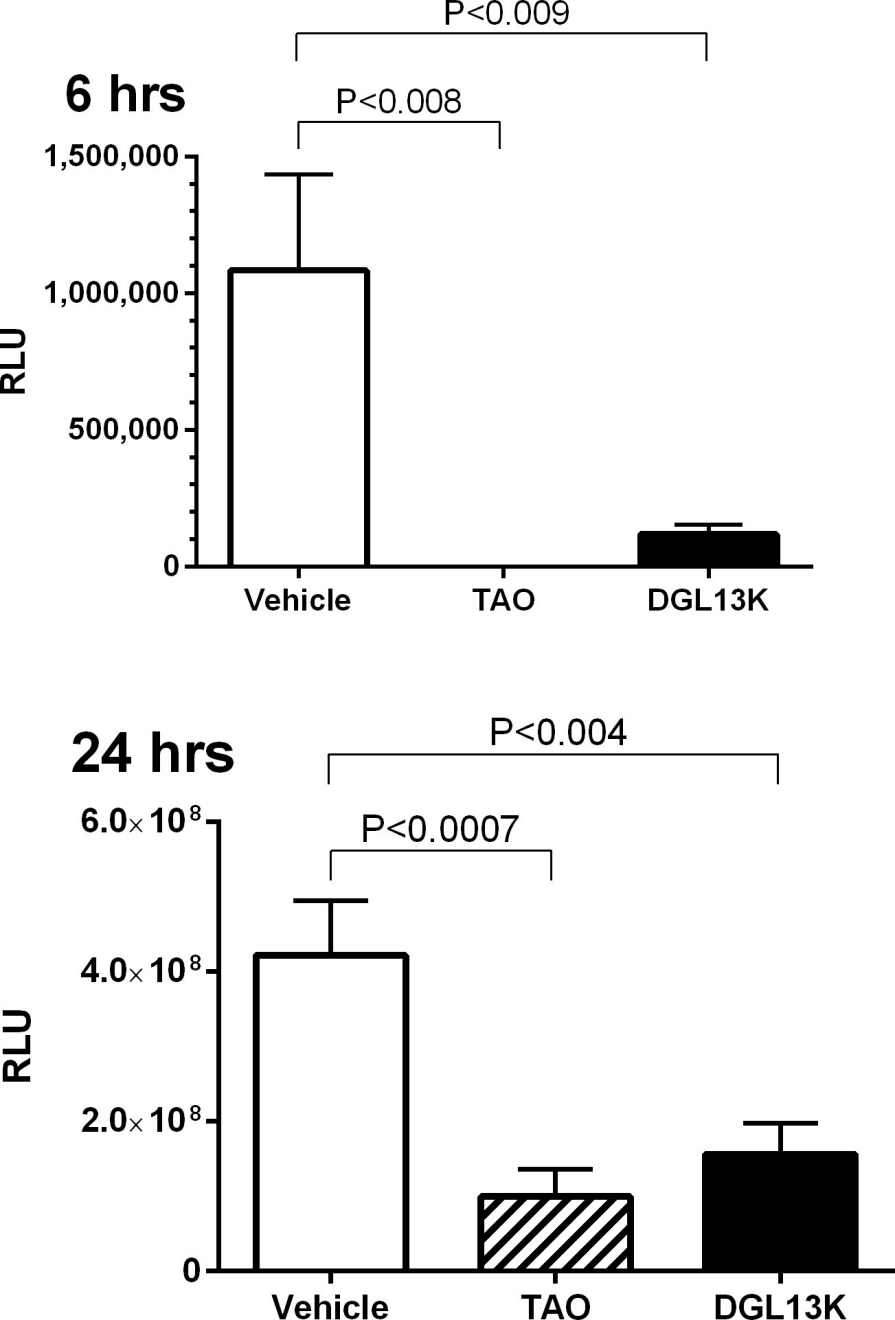
Mouse skin burn wound infection model. Infected mice were treated with HPC vehicle (Vehicle), triple antibiotic ointment (TAO) or DGL13K in HPC vehicle (DGL13K), as described in Fig 6. The mice were imaged at 6 hours (left panel) and 24hours (right panel) after inoculation to determine RLU emitted by remaining live bacteria. The experiment was performed twice with 5 mice per group. Data are shown as mean±SEM after removal of statistical outliers (N = 7–10). Antibiotic treatment groups were compared to the vehicle control by one-way ANOVA with Dunnet’s multiple comparison post-test.

The efficacy of topical DGL13K was tested in a mouse burn wound infection model, adapted from published reports [25, 26]. A preliminary experiment showed that a 2 sec burn with a brass rod heated to 95°C created a second-degree burn with loss of epithelium and some effect on dermal layers (Fig 6B). A longer burn (4 sec) created a deep 2/3° burn with loss of both epithelial and dermal layers (Fig 6C). The second-degree burn was selected as the most clinically relevant for topical antibiotic treatment. Burn wounds were inoculated with 10^5^ CFU *P*. *aeruginosa* Xen 41 per mouse. The mice were treated topically with DGL13K ointment or triple antibiotic ointment 2 hours and 6 hours after inoculation, with imaging 6 hours and 24 hours after inoculation, i.e. 4 hours and 18 hours after the two topical treatments, respectively (Fig 6D). Both treatments reduced the load of live bacteria about 10-fold at 4 hours after the first treatment and about 4-fold at 18 hours after the second treatment, compared to vehicle controls (Fig 7).

## Discussion

The second generation AMP DGL13K shows promising antibacterial properties against both Gram negative [13, 14] and Gram positive bacteria [15], including bacterial biofilms and drug-resistant bacteria [14, 15] (this report). Notably, repeated use of DGL13K at sub-inhibitory concentrations does not lead to bacterial resistance [15], an important advantage for a potential antibiotic.

To further evaluate the antibiotic potential of DGL13K, aspects of peptide toxicity and activity were tested in two cell culture and two animal models. Cellular toxicity of DGL13K was observed at concentrations above about 250 μg/ml and LD50 was reached at about 1 mg/ml in both human erythrocytes and HEK cells. Combined with an MIC of 5 μg/ml, this suggests a therapeutic index (LD50/MIC) of 200, which compares favorably with the activity and kidney cell cytotoxicity reported for a novel derivative of polymyxin [27, 28].

The low toxicity observed in human cells was reflected in the absence of skin toxicity after topical application of DGL13K (compare Fig 4D and 4E and Fig 6A). In contrast, DGL13K was more toxic in the *G*. *mellonella* injection model. Time course experiments showed that toxicity occurred early after injection. Larvae that survived the initial toxicity, generally survived for 24 hours. The dose causing 50% mortality in the larvae was about 25-fold higher than the MIC of DGL13K against *P*. *aeruginosa*.

The use of fluorescent bacteria for high-throughput screening of bacterial virulence in vivo has recently been reported [22]. We used a variation of this method that combines infection of *G*. *mellonella* with bioluminescent bacteria in a high-throughput format. Unlike the previous report [22], we used optically clear polystyrene plates for up to 24h with no adverse effects from larval feeding. In addition to monitoring bacterial growth in real-time in live larvae, we also monitored the inhibition of bacterial growth by DGL13K in vivo. The ability to perform kinetic analyses in 24 larvae simultaneously, greatly increases the number of conditions that can be tested at minimal cost compared to more traditional mammalian models. Thus, the *G*. *mellonella* infection model is an ideal intermediate step between cell culture and more resource intensive mammalian infection models.

In addition to biological activity in the *G*. *mellonella* model, DGL13K reduces bacterial load in a mouse burn wound infection model. The peptide reduces bacterial load about 10-fold after 6 hours, similar to the effect of the triple antibiotic ointment used as a control. Both treatments remain effective for at least 24 hours, although the overall effect is reduced to about 3-fold reduction of bacterial load. Future experiments will focus on optimizing peptide delivery, dose and frequency to improve the antibacterial effect further.

## Abbreviations

AMP: antimicrobial peptide
CFU: colony-forming units
HEK: HEK-Blue hTLR4
HPC: hydroxy-propyl cellulose
LD50: median lethal dose
LDH: lactate dehydrogenase
MIC: minimal inhibitory concentration
OD: optical density
PBS: phosphate-buffered saline
RBC: red blood cells
RLU: relative light units
THB: Todd Hewitt Broth
